# The survival of blown pack spoilage associated *Clostridium estertheticum* and *Clostridium gasigenes* spores during the ensiling of grass

**DOI:** 10.1093/femsmc/xtab013

**Published:** 2021-10-02

**Authors:** Eden Esteves, Paul Whyte, Tanushree B Gupta, Declan Bolton

**Affiliations:** Department of Food Safety, Teagasc Food Research Centre, Ashtown, Dublin 15, Ireland; School of Veterinary Medicine, University College Dublin, Belfield, Dublin 4, Ireland; Food Assurance Team, AgResearch Ltd, Hopkirk Research Institute, Massey University, Palmerston North 4472, New Zealand; School of Veterinary Medicine, University College Dublin, Belfield, Dublin 4, Ireland; Food Assurance Team, AgResearch Ltd, Hopkirk Research Institute, Massey University, Palmerston North 4472, New Zealand; Department of Food Safety, Teagasc Food Research Centre, Ashtown, Dublin 15, Ireland

**Keywords:** *Clostridium estertheticum*, *Clostridium gasigenes*, ensiling, silage, blown pack spoilage

## Abstract

Blown pack spoilage (BPS) of vacuum packaged primals, caused by *Clostridium estertheticum* and *Clostridium gasigenes*, is a serious issue for the beef industry. There are multiple sources of these bacteria on beef farms, including grass and associated feed preparations. The aim of this study was to investigate the survival of *C. estertheticum* and *C. gasigenes* spores during the ensiling of grass and the subsequent opening of the silos. Grass, harvested from fields, with and without cattle slurry amendment, was inoculated with approximately 100 spores/g and ensiled using a laboratory (silo) model system at 20°C in the dark. Adding formic acid or sucrose resulted in six treatment combination as follows: no slurry (NS), no slurry plus formic acid (NSFA), no slurry plus sucrose (NSS), slurry (S), slurry plus formic acid (SFA) and slurry plus sucrose (SS). During the silage fermentation, samples were removed periodically and tested for *C. estertheticum*, *C. gasigenes*, total viable, *Escherichia coli*, *Enterobacteriaceae* and lactic acid bacteria (LAB) counts. The pH, ethanol, volatile fatty acids (VFA), lactic acid and ammonia concentrations were also monitored throughout the experiment. *C. estertheticum* did not survive the ensiling process, regardless of treatment. In contrast, *C. gasigenes* grew in the early stages and was detected during the entirety of the fermentation for all treatments. Based on these observations, it was concluded that the silage fermentation process described would not remove *C. gasigenes* and contaminated grass may result in contaminated feed for animals.

## INTRODUCTION

Blown pack spoilage (BPS) of chilled vacuum packaged beef, primarily caused by *Clostridium estertheticum* and *Clostridium gasigenes* (Moschonas *et al*. 2010) is a major issue for the meat industry (Brightwell, Broda and Boerema 2009). These bacteria are widespread in the farming environment including on the grass used for the preparation of silage (Esteves *et al*. 2020). Survival and/or growth during the silage fermentation process could expose animals to ingestion of large numbers of BPS *Clostridium* spores, potentially contaminating faeces, hides, carcasses and ultimately vacuum packaged primal cuts (Bolton, Carroll and Walsh 2015). BPS of beef occurs in correctly chilled vacuum packaged batches (0–4°C) after 4-6 weeks and is characterized by the production of large volumes of gas, a putrid smell with a metallic sheen on the meat. The spoiled meat is unacceptable for human consumption and has no commercial value (Cavill *et al*. 2011; Clemens *et al*. 2010).

Silage is grass preserved by fermentation (Muck, Moser and Pitt 2003). The ensiling process may be divided into three stages; (1) harvesting, chopping and packing resulting in cell respiration that produces carbon dioxide, heat and water, until either the oxygen supply or water-soluble carbohydrates are depleted; (2) anaerobic hetero-fermentation, mainly by heat tolerant *Enterobacteriaceae*, lactic acid bacteria (LAB) and other anaerobes at pH 5.0–7.0, that compete for available substrates, with acetic and lactic acid being the main fermentation products, and (3) faster growing LAB species dominate towards the end of ensiling and lactic acid is the main fermentation product, resulting in a decrease in pH to approximately pH 4.0–4.5. During storage the temperature usually ranges from 25 to 35°C and the grass is preserved by the low pH and anaerobic conditions. However, once the silage pit or bags are opened aerobic decomposition by moulds and yeast may occur (Weinberg *et al*. 2010).

Carbohydrate, proteins and/or lactic acid may be used as energy sources by *Clostridium* species (Cremonesi *et al*. 2012; Driehuis, Hoolwerf and Rademaker 2016). Previous studies suggest these bacteria may grow during silage fermentation or survive as spores, if there is sufficient moisture and mild pH conditions, due to inadequate wilting of the grass, rain or seepage (Muck, Moser and Pitt 2003). Moreover, *Clostridium* spp. spores, including *C estertheticum* and *C. gasigenes* have been previously detected in farm silage samples (Esteves *et al*. 2020).

Cattle slurry is the main fertilizer applied to grass on Irish farms pre-harvest to promote growth. During silage preparation, grass may be supplemented with formic acid or sucrose to induce a drop in pH to produce good quality silage (Li *et al*. 2014; Ren *et al*. 2018). The aim of this study was to investigate the survival of *C. estertheticum* and *C. gasigenes* during the ensiling of grass as undertaken on Irish farms (harvested from fields with and without cattle slurry amendment and ensiled with and without formic acid and sucrose) using a laboratory model system at 20°C.

## MATERIALS AND METHODS

### Bacterial strains and preparation of spore inoculum

Type strains of *C. estertheticum subsp. estertheticum* (strain DSMZ 8809^T^, T = type strain) and *C. gasigenes* (DSMZ 12272^T^) were purchased from Deutsche Sammlung von Mikroorganismen und Zellkulturen GmbH (DSMZ, Braunschweig, Germany). These reference strains were cultivated anaerobically for >14 days at 10°C and 20°C, respectively, in pre-reduced peptone yeast extract glucose starch (PYGS; Sigma Aldrich, Wicklow, Ireland) broth and stored at 4°C until further use. Spore concentrates of *C. estertheticum* and *C. gasigenes* were prepared by transferring 5 mL of exponentially growing culture (up to 1–2 months) to 100 mL of pre-reduced PYGS broth and incubating at 4°C for a minimum of 3 months to promote sporulation (Reid *et al*. 2017). Prior to inoculation all sterilized media were cooled and stored inside an anaerobic cabinet (Don Whitley Scientific Ltd, Shipley, UK), under an atmosphere of mixed gas, CO_2_ and N_2_ at 37°C and used within 48 h. Spores were harvested using the method described by (Reid *et al*. 2017). Briefly, spore suspensions were recovered by centrifugation (7500 × *g*, 4°C, 10 min) and washed with saline (0.85% NaCl; Sigma Aldrich) three times. The washed spore suspension was then sonicated (40 kHz for 15 min) in an ultrasonic water-bath (VWR International, Dublin 15, Ireland) at room temperature. Sonication/centrifugation/wash cycles were repeated three times. The spores were then suspended in 10 mL saline and stored at –20°C. Final spore numbers were enumerated by preparing 10-fold serial dilutions of the heat treated (80°C, 10 min) spore suspensions in saline and plating out 0.1 mL aliquots on Columbia blood agar (CBA; CM0331, Oxoid Ltd, Basingstoke, UK) supplemented with 5% defibrinated horse blood and incubated anaerobically for 3 weeks at 4°C.

### Preparation of grass crops and harvest

Three blocks within a permanent sward of perennial ryegrass (*Lolium perenne* L.) at Teagasc Beef Research Centre (Grange, County Meath, Ireland) were each subdivided into six plots (5 m × 1 m each). Based on random selection, each block was halved, and cattle slurry (collected from a slatted floor tank in a roofed cattle shed on the same research farm) was manually applied at the equivalent of 30 tonnes/ha. After approximately 7 weeks growth, the grass was harvested using a lawn mower cutting to a stubble height of approximately 5 cm. The mower was run in a plot harvest sequence to avoid cross contamination between the slurry-treated and the non-slurry plots. The following treatment combinations were applied to grass from the six plots; no cattle slurry (NS), no cattle slurry plus formic acid (NSFA), no cattle slurry plus sucrose (NSS), cattle slurry applied (S), cattle slurry plus formic acid (SFA) and cattle slurry plus sucrose (SS). Appropriately, 2.5 mL formic acid or 25 g of sucrose was applied to per kg of grass shortly before packing grass into tubes.

### Dry matter concentration analysis

Empty 2 L Pyrex glass beakers were weighed. Duplicate samples from each plot pre-treatment (NS and S) were placed in this these beakers, weighed and dried at 98°C for 16 h in an oven with forced-air circulation, reweighed and the dry matter concentration calculated as described by Nyambe *et al*. (2017).

### Inoculation of grass samples

Samples from within each treatment combination within each block were inoculated to a final concentration of approximately 100 spores of *C. estertheticum* or *C. gasigenes* per g of grass or left uninoculated. Exactly, 65 g of the inoculated and un-inoculated samples were individually packed into 100-mL glass test tubes, at a packing density of 0.65 or 65% (mass of the grass in the tube divided by the volume of the tube) and fitted with a rubber stopper through which a water filled gas release valve was placed. These packed silos were stored in a laboratory incubator, in the dark, at 20°C. Replicate tubes (x3) were opened at 10 sampling times, *t* = 0, 11, 22, 33, 44, 55, 66, 77 and 88 days after which the remaining test tubes were left unplugged for a further 11 days to mimic opening of the silage pit (these tubes were analysed at *t* = 99). The contents from inoculated and uninoculated tubes were microbiologically and chemically analysed.

### Chemical analysis

Chemical analysis was carried out on an aqueous extract obtained from each silage treatment prepared using uninoculated grass samples. Each aqueous extract was analysed for pH (Eutech, pH5+ Meter, Dublin, Ireland), using a handheld specific electrode which was calibrated with specific pH 4.0 and pH 10.0 standards. Each aqueous extract was also assayed for lactic acid and ammonia at 340 nm (D-/L- Lactic acid assay (Rapid) (Nzeteu *et al*. 2018) and Ammonia assay (Rapid; Lee, Chung and Kim 2012) kit, Megazyme, Wicklow, Ireland) by using the single point standard microplate assay procedure as per the manufacturer's instructions. The concentrations of lactic acid and ammonia were calculated using the following formula:

$$\begin{eqnarray*}
\frac{{\Delta\,Absorbance\;in\;a\;sample}}{{\Delta\, Absorbance\;in\;the\;standard}} \times g/{L} \times dilution\;factor.\end{eqnarray*}$$

The ethanol and volatile fatty acid (VFA) content in uninoculated samples was determined by gas chromatography coupled to a flame ionization detector (Varian GC CP 3800 with Combi Pal; Hong *et al*. 2015).

### Microbiological analysis

The contents from each lab-silo were carefully placed into stomacher bags and homogenized with 100 mL of maximum recovery diluent; (MRD CM0733B, Oxoid Ltd, Basingstoke, UK) using a stomacher for 1 min. Serial dilutions of the resulting suspension were spread-plated in duplicate. Total viable counts (TVC) were enumerated on plate count agar (PCA CM0325, Oxoid Ltd), incubated at 30°C for 48 h. Total Enterobacteriaceae counts (TEC) were obtained using violet red bile glucose agar; (VRBGA CM0485, Oxoid Ltd) incubated at 37°C for 24 h. *Escherichia coli* counts (EEC) were enumerated on Eosin methylene blue agar (EMB CM0069, Oxoid Ltd) and incubated for 24 h at 37°C, while LAB were enumerated by plating on De Man, Rogosa and Sharpe; (MRS CM0361, Oxoid Ltd) and incubated at 30°C for 48–72 h. For the enumeration of *C. estertheticum* and *C. gasigenes*, samples were plated on Columbia blood agar; (CBA CM0331, Oxoid Ltd) and incubated at 4°C for 3 weeks. *Clostridium estertheticum* colonies were round with often coarsely granulated margins, smooth, slightly raised, cream-white to greyish and semi-transparent to opaque and non-haemolytic, while, *C. gasigenes* colonies appeared as grey–white and opaque, circular, raised, convex, shiny and smooth β-haemolytic colonies.

### Confirmation of *C. estertheticum* and *C. gasigenes*

To confirm that the presumptive colonies were *C. estertheticum* and *C. gasigenes*, four-five colonies were randomly selected from each CBA plated sample, suspended in 1 mL phosphate buffered saline (PBS, Oxoid Ltd, Basingstoke, UK) and centrifuged at 5000 × *g* for 10 min. The resultant pellet was washed with 1 mL PBS before 10 mg/mL lysozyme was added and incubated at 37°C for 30–60 min to lyse the cells. DNA was extracted using a DNeasy Blood and Tissue Kit (Qiagen Ltd, Crawley, UK) according to manufacturer's protocol for the isolation of Gram positive bacteria. Quantitative real time PCR (qPCR) was performed using a LightCycler 480 platform (LC480; Roche Diagnostics GmbH) as per the method of (Reid *et al*. 2017). TMF (forward primer), TMR (reverse primer) and Probe were used for the detection of *C. estertheticum*. 16SDB_for (forward primer), 16SDB-A (reverse primer) and 16sDB_TM probe was used for the detection of *C. gasigenes* (Table 1). All primers and probes used were purchased from Tib Molbiol, Berlin, Germany. For the *C. estertheticum* assay the qPCR was carried out in a 10 µL reaction volume containing 0.3 µmol/L primer and 0.1 µmol/L probe, 2.8 µL H_2_O, 5 µL LightCycler 480 Probe master mix (2X; Roche Diagnostics) and 1 µL of DNA to be tested. A positive (*C. estertheticum*) and negative (*C. gasigenes*) DNA control and no template control (NTC) were included in each qPCR run. The cycling protocol included an initial stage at 95°C for 10 min, followed by 45 cycles (95°C for 10 s, 65°C for 30 s and 72°C for 1 s). For *C. gasigenes* the qPCR was performed in a 10 µL reaction volume containing 0.5 µmol/L primer and 0.2 µmol/L probe, 2.8 µL H_2_O, 5 µL LightCycler 480 Probe master mix (2X; Roche Diagnostics) and 1 µL of DNA to be tested. A positive (*C. gasigenes*) and negative (*C. estertheticum*) DNA control and no template control (NTC) were included in each run. The cycling protocol included an initial stage at 95°C for 10 min, followed by 45 cycles (95°C for 10 s, 62°C for 30 s and 72°C for 1 s). The concentration of *C. estertheticum* and *C. gasigenes* was determined by using appropriate standards with known concentrations in qPCR during each run.

**Table 1.** Primer and probe sequences used for the detection of *C. estertheticum* and *C. gasigenes*.

*Clostridium* spp.	Primer name	Primer sequence
*C. estertheticum*	TMF (forward primer)	5′ CGG CGG ACG GGT GAG TAA C 3′
	TMR (reverse primer)	5′ CGG GTC CAT CTC AAA GTG RAA CT 3′
	Probe	5′ FAM- CGT GGG TAA CCT GCC TCA AAG AGG GG-TAMARA 3′
*C. gasigenes*	16SDB_for (forward primer)	5′ GAG AGG AGT TCT TCG GAA CGA 3′
	16SDB-A (reverse primer)	5′ GGA TTT CTC CTT TAA TTG CTG CT 3′
	16sDB_TM Probe	5′ FAM-ATG CGA AAC TGC AAT GTT ATG CGGT—Q 3′

### Statistical analysis

The experiment was performed using x3 replicates of the six different treatment combinations of × 2 (no slurry + cattle slurry application) × 2 (formic acid and cattle slurry + formic acid treatment during ensiling of grass) × 2 (sucrose and cattle slurry + sucrose treatment during ensiling of grass). Data analysis of the inoculated grass samples was conducted using a two way analysis of variance (ANOVA) test to determine the F-statistic (to compare initial bacterial counts with subsequent sampling points) and a post-hoc tukey test to compare means using GraphPad Prism 7.02 software (GraphPad Software, San Diego, CA, USA).

## RESULTS

### Dry matter concentration analysis

The recorded values of dry matter (DM g/kg) for the pre-treated herbage (NS and S) were 222 g/kg and 212.5 g/kg, respectively.

### pH measurement

In the NS test-tube silos, the pH decreased from 5.4 to 4.0 after 11 days, increased to 4.8 (day 44), decreasing to 4 (55–88 days) and increased to pH 5.2 on day 99 (Table 2). The NSFA fermentation showed a decrease in pH from 5.4 to 3.9 after 22 days and remained at approximately this pH until day 99, when a pH of 5.4 was recorded. A similar pattern was observed with the NSS fermentation although the initial and final pH values (both 6.4) were higher than NSFA. The slurry pre-treatment of the grass appeared to inhibit the fermentation process, although a slight (0.5 pH units) decrease was observed, the pH values remained between 5.2 and 5.8 in the S test-tube silos. The addition of formic acid to slurry pre-treated grass (SFA) lowered the initial pH to 5.3 but the fermentation was again inhibited, as the lowest pH achieved was 4.4 after 88 days. Replacing formic acid with sucrose (SS) resulted in a steady decrease in pH from 5.8 to 4.1 after 44 days, the pH stayed at 4.1 until day 88 after which there was an increase to 5.8 recorded by day 99.

**Table 2.** pH values over the course of the ensiling process with the different treatment in the laboratory model silos.

	Treatment					
Time (d)	NS	NSFA	NSS	S	SFA	SS
0	5.8	5.4	6.4	5.8	5.3	5.8
11	4	5.5	5.2	5.7	5.1	5.4
22	4.1	3.9	4.2	5.5	4.9	5.1
33	4.6	4.1	4.3	5.2	4.9	4.3
44	4.8	4	4.3	5.3	4.6	4.4
55	4	3.9	4.3	5.3	4.6	4.2
66	3.9	4	4.3	5.4	4.6	4.1
77	3.9	4.1	4.1	5.5	4.5	4.1
88	3.9	4	3.9	5.2	4.4	4.1
99	5.2	5.4	6.4	5.8	5.3	5.8

### Chemical analysis

The concentrations of ethanol, acetic acid, propionic acid, n-butyric acid and lactic acid are provided in Tables 3 and 4. The highest levels of ethanol were found in NSS and SS treatment ranging from 54.1 to 106 g/L between 33 and 66 days. Acetic acid concentrations ranged from approximately 0.1 to 7.8 g/L regardless of treatment. Propionic acid was found in low levels ranging from 0.1 to 1.7 g/L regardless of treatment. *n*-Butyric acid concentrations remained <3.8 g/L regardless of treatment throughout ensiling. Lactic acid concentration increased for all treatment combinations ranging from 10 to 76 (NS), 7 to 77 (NSFA), 15 to 55 (NSS), 10 to 186 (S), 6 to 136 (SFA) and 26 to 219 (SS) g/L. Reduced levels of lactic acid were recorded after the silos were left open. Ammonia concentrations ranged from 0.02 to 0.1 (data not shown), regardless of the treatment or sampling time.

**Table 3.** Ethanol and VFA concentrations (g/L) of un-inoculated samples recorded during the ensiling of grass treated with NS, NSFA and NSS.

	Treatment														
	NS					NSFA					NSS				
Time (d)	Ethanol and VFA's (g/L)														
	E	AA	PA	BA	LA	E	AA	PA	BA	LA	E	AA	PA	BA	LA
0	12.2	4.0	0.4	0.1	19	10.7	4.1	0.5	0.0	7	13.0	3.7	0.1	0.0	15
11	10.2	3.0	0.6	0.0	26	3.6	0.9	0.2	0.2	17	2.3	0.3	0.1	0.0	21
22	17.8	3.0	0.3	0.0	27	25.2	4.2	1.7	0.3	30	24.7	7.4	1.6	0.6	29
33	5.4	1.7	0.3	1.2	27	2.0	3.8	1.1	0.9	37	15.8	1.1	0.1	0.1	38
44	4.6	3.8	0.7	1.6	25	10.8	3.5	0.7	0.5	45	54.1	4.9	0.8	1.2	52
55	10.5	3.3	0.7	0.2	26	10.5	3.6	0.9	1.0	56	106.1	5.6	0.5	0.5	53
66	3.3	5.9	0.7	0.2	33	8.7	2.0	0.9	1.6	71	92.0	2.2	0.4	0.4	65
77	2.7	0.2	0.1	1.0	49	8.2	1.5	0.8	1.1	71	36.2	4.4	0.7	0.5	66
88	1.6	0.1	0.0	0.1	76	8.4	1.3	0.2	0.2	77	9.5	2.7	0.4	0.6	55
99	1.1	0.0	0.0	0.0	51	7.3	1.2	0.2	0.4	18	1.7	0.4	0.3	0.5	19

**Table 4.** Ethanol and VFA concentrations (g/L) of un-inoculated samples recorded during the ensiling of grass treated with S, SFA and SS.

	Treatment														
	S					SFA					SS				
Time (d)	Ethanol and VFA's (g/L)														
	E	AA	PA	BA	LA	E	AA	PA	BA	LA	E	AA	PA	BA	LA
0	1.8	1.0	0.0	0.0	10	7.5	1.4	0.0	0.0	6	13.7	3.7	0.4	0.0	26
11	6.5	4.5	1.7	3.8	16	3.1	0.9	0.1	0.1	12	36.9	3.1	0.2	0.1	36
22	5.7	4.4	1.5	2.3	30	0.9	0.4	0.1	0.0	14	47.1	2.9	0.2	0.2	37
33	1.5	2.8	1.0	1.4	35	1.4	1.5	0.3	0.7	16	60.8	0.3	0.0	0.1	45
44	2.8	5.4	2.0	2.0	42	3.7	3.7	1.1	1.2	19	67.2	7.8	0.9	0.7	63
55	7.0	6.6	2.8	3.1	51	8.2	2.3	0.6	1.8	50	23.0	5.3	1.0	0.2	133
66	14.7	5.0	2.6	3.6	65	2.9	0.5	0.3	0.7	70	19.0	1.8	0.8	4.6	161
77	3.3	3.7	2.1	3.5	123	11.0	1.7	0.6	2.2	87	7.1	0.4	0.1	0.2	187
88	4.0	3.3	1.7	3.2	186	1.8	1.6	0.6	2.6	136	2.3	0.2	0.1	0.1	219
99	4.1	2.3	1.4	2.7	46	1.5	1.1	0.5	1.3	15	1.0	0.2	0.1	0.3	47

### Microbiological analysis

The initial TVC ranged from 4.9 to 6.3 log_10_ CFU/g, with significantly (*P* < 0.05) higher counts on samples from NS, NSFA and SS samples as compared to NSS, S and SFA samples (Table 5). By day 11, all counts had increased to between 7.4 and 8.6 log_10_ CFU/g and by day 33 had further increased between 8.0 and 9.1 log_10_ CFU/g for all treatments. Thereafter the counts remained within 2 log_10_ CFU/g of these values until the end of the study (99 days). Although statistical differences were observed between different treatments and at different times, there was no overall treatment that consistently resulted in higher or lower counts. The initial TEC ranged from 2.7 to 5.2 log_10_ CFU/g and there was no evidence that higher counts were obtained on grass samples harvested from fields pre-treated with slurry. Maximum TEC of 5.6, 6.0, 5.2, 7.0, 5.7 and 4.8 were obtained after 11–33 days on NS, NSFA, NSS, S, SFA and SS samples, respectively. Thereafter TEC decreased significantly reduced after 44–77 days. As with the TVC, there was no treatment that consistently resulted in significantly (*P* < 0.05) higher or lower counts as compared to the other treatments. Initial ECC of 2.4–4.8 log_10_ CFU/g increased to maximum values of 4.1–5.7 log_10_ CFU/g after 33–55 days, thereafter, decreasing to undetectable levels after 66–77 days, regardless of treatment. No treatment consistently resulted in significantly (*P* < 0.05) higher or lower counts throughout the study.

**Table 5.** Total viable, *Enterobacteriaceae*, *E. coli* and LAB counts during ensiling of grass with the different treatments.

	NS		NSFA		NSS		S		SFA		SS	
Time (d)	TVC (log_10_ CFU/g)											
	Mean	SE	Mean	SE	Mean	SE	Mean	SE	Mean	SE	Mean	SE
0	6.3^A^	0.4	5.8^A^	0.1	5.0^B^	0.5	4.9^B^	0.0	5.0^B^	0.5	5.8^A^	0.8
11	7.8^AB^	0.2	7.4^B^	0.9	8.5^A^	0.2	8.3^A^	0.0	8.6^AB^	0.2	7.5^B^	0.9
22	8.1^A^	0.3	8.4^A^	0.3	7.9^A^	0.6	7.8^A^	0.0	8.4^A^	0.3	7.8^A^	0.4
33	8.8^AB^	0.2	9.1^A^	0.6	8^B^	0.2	8.6^AB^	0.1	8.8^AB^	0.2	9.1^A^	0.0
44	8.1^A^	0.6	7.4^B^	0.4	7.4^B^	0.2	7.8^AB^	0.4	8.0^A^	0.4	8.3^A^	0.0
55	7^A^	0.5	7.3^A^	0.6	8.4^B^	0.2	7.3^AB^	0.8	8.5^B^	0.1	8.1^B^	0.3
66	8.0^AB^	0.4	7.1^B^	0.7	8.3^A^	0.3	7.4^B^	0.7	8.2^AB^	0.1	8.2^AB^	0.0
77	7.4^AB^	0.2	7.2^B^	0.5	8.0^A^	0.1	6.9^B^	0.5	7.7^AB^	0.1	7.4^AB^	0.3
88	6.8^A^	0.3	6.8^A^	0.2	6.7^A^	1.1	5.6^B^	0.3	7.0^A^	0.2	6.9^A^	0.0
99	6.9^A^	0.4	7.4^A^	0.1	6.9^A^	0.1	7.0^A^	0.0	7.2^A^	0.2	7.4^A^	0.3
	TEC (log_10_ CFU/g)											
0	4.6^AB^	0.8	2.7^C^	0.4	4.1^B^	0.8	5.2^A^	1.2	3.8^B^	1.1	4.7 ^AB^	0.1
11	5.6^A^	0.4	4.9^B^	1.1	3.9^B^	0.6	6.5^A^	0.4	5.7^A^	0.4	4.8^B^	1.0
22	3.9^C^	1.4	5.1^B^	0.1	4.5^BC^	1.1	4.5^BC^	0.1	5.6^A^	0.2	3.8^C^	0.8
33	3.7^D^	1.2	6.0^B^	0.6	5.2^BC^	0.5	7.0^A^	0.3	5.6^BC^	0.2	4.8^C^	0.1
44	2.3^B^	0.1	0.0^C^	0.0	4.3^A^	0.2	5.2^A^	0.1	4.5^A^	0.3	4.2^A^	0.2
55	2.0^B^	0.0	0.0^C^	0.0	2.1^B^	0.2	0.0^C^	0.0	4.5^A^	0.4	0.0^C^	0
66	0.0^A^	0.0	0.0^A^	0.0	0.0^A^	0.0	0.0^A^	0.0	3.5^B^	0.2	0.0^A^	0
77	0.0^A^	0.0	0.0^A^	0.0	0.0^A^	0.0	0.0^A^	0.0	0.0^A^	0.0	0.0^A^	0
88	0.0^A^	0.0	0.0^A^	0.0	0.0^A^	0.0	0.0^A^	0.0	0.0^A^	0.0	0.0^A^	0
99	0.0^A^	0.0	0.0^A^	0.0	0.0^A^	0.0	0.0^A^	0.0	0.0^A^	0.0	0^A^	0
	*E. coli* counts (log_10_ CFU/g)											
0	4.4^AB^	0.2	2.4^C^	1.0	3.1^C^	0.2	4.8^AB^	0.0	4.1^B^	0.4	4.5^AB^	0.1
11	5.2^A^	0.6	4.7^B^	0.0	3.9^B^	0.4	4.4^AB^	0.4	4.8^B^	0.0	4.5^AB^	0.2
22	4.9^A^	0.5	4.4^AB^	0.5	4.1^AB^	0.5	3.5^B^	0.4	4.9^AB^	0.1	5^A^	1.0
33	5.3^A^	0.3	5.7^AB^	0.2	3.0^BC^	0.2	4.8^AB^^C^	0.2	4.3^B^	0.5	3.7^C^	0.4
44	5.2^AB^	0.2	4.3^AC^	0.1	3.8^C^	1.1	4.2^C^	0.1	5.7^AB^	0.1	5.4^B^	0.4
55	0.0^C^	0.0	5.4^AB^	0.2	0.0^C^	0.0	4.1^B^	0.9	5.6^AB^	0.2	5.5^AB^	0.9
66	0.0^B^	0.0	1.9^B^	0.1	0.0^B^	0.0	0.0^B^	0.0	4.7^A^	0.6	0.0^B^	0.0
77	0.0^A^	0.0	0.0^A^	0.0	0.0^A^	0.0	0.0^A^	0.0	0.0^A^	0.0	0^A^	0.0
88	0.0^A^	0.0	0.0^A^	0.0	0.0^A^	0.0	0.0^A^	0.0	0.0^A^	0.0	0^A^	0.0
99	0.0^A^	0.0	0.0^A^	0.0	0.0^A^	0.0	0.0^A^	0.0	0.0^A^	0.0	0^A^	0.0
	LAB (log_10_ CFU/g)											
0	4.1^A^	0.0	4.1^A^	0.0	4.1^A^	0.0	4.3^A^	0.3	4.7^A^	0.8	4.1^A^	0.0
11	7.8^A^	0.2	7.4^A^	0.9	8.5^A^	0.3	7.9^A^	0.5	8.0^A^	0.3	8.1^A^	0.2
22	7.8^A^	0.3	7.8^A^	0.4	7.9^A^	0.4	7.2^A^	0.4	8.0^A^	0.1	7.3^A^	1.0
33	8.0^A^	0.4	7.6^A^	0.4	7.0^A^	0.8	7.7^A^	0.4	7.8^A^	0.2	7.6^A^	0.1
44	7.7^A^	0.3	7.1^AB^	0.1	7.2^AB^	0.6	7.4^AB^	0.3	6.8^B^	0.2	7.6^AB^	0.5
55	7.1^A^	0.1	6.2^A^	0.4	6.4^A^	0.5	6.4^A^	1.0	7.6^A^	0.4	7.2^A^	0.1
66	7.5^A^	0.4	6.9^A^	1.1	7.6^A^	0.5	6.5^A^	0.5	7.0^A^	0.6	7.0^A^	0.9
77	6.0^A^	0.4	4.9^B^	3.5	6.2^A^	0.5	6.3^A^	0.5	7.1^A^	0.1	7.1^A^	0.3
88	5.4^A^	0.6	3.8^B^	2.7	6.0^A^	0.4	4.7^A^	0.4	6.3^A^	0.3	5.7^A^	0.3
99	6.2^A^	0.3	6.1^A^	0.1	5.9^A^	0.5	6.3^A^	0.1	5.7^A^	0.2	6.2^A^	0.2

SE, standard error.

ABCD different superscript letters denotes statistical difference (*P* < 0.05) between all treatment combinations at a given time (*t* = 0–99 days).

LAB are responsible for fermenting the grass during the ensiling process and as expected increased from approximately 4.0–8.0 log_10_ CFU/g after approximately 11 days, remaining at this concentration up to 44 days and thereafter maintaining a concentration of approximately 4.0–7.5 log_10_ CFU/g, regardless of treatment.

*C. estertheticum* and *C. gasigenes* spores were inoculated onto the grass to a mean concentration of 2.1 log_10_ CFU/g. *C. estertheticum* did not survive the ensiling process and were not detectable in any of the samples after 22–44 days (Table 6). Prior to this, the counts of *C. estertheticum* in the SS silos were significantly (*P* < 0.05) higher than in any other treatment while there was no clear pattern for the other treatments. In contrast, *C. gasigenes* survived for the entirety of the ensiling process, regardless of treatment (Table 7). Moreover, growth was observed in four of the treatments, reaching maximum values of 5.4, 6.7, 3.6 and 3.8 log_10_ CFU/g in NS, NSS, S and SS, silos respectively after 11–33 days. Thereafter the counts in all silos were approximately 1–2 log_10_ CFU/g, with final counts after 99 days ranging from 1.0 to 1.3, log_10_ CFU/g. No treatment consistently achieved significant higher or lower counts when compared to other treatments. By days 88 and 99, all of the *C. estertheticum* counts were statistically similar regardless of treatment.

**Table 6.** Changes in the concentration of *C. estertheticum* (from the mean inoculation level of 2.1 log_10_ CFU/g), during the ensiling process with the different treatments.

	NS		NSFA		NSS		S		SFA		SS	
Time (d)	*C. estertheticum* (log_10_ CFU/g)											
	Mean	SE	Mean	SE	Mean	SE	Mean	SE	Mean	SE	Mean	SE
11	0.4^D^	0.3	1.6^C^	0.2	0.6^CD^	0.3	2.6^B^	0.1	1.2^C^	0.1	3.4^A^	0.2
22	1.0^B^	0.9	0.3^C^	0.1	ND^C^	0.0	1.6^B^	0.2	ND^C^	0.0	2.3^A^	0.2
33	2.1^B^	1.2	2.5^B^	0.2	ND^C^	0.0	ND^C^	0.7	ND^C^	0.0	3.3^A^	0.1
44	ND*^A^	0.0	ND^A^	0.0	ND^A^	0.0	ND^A^	0.0	ND^A^	0.0	ND^A^	0.0
55	ND^A^	0.0	ND^A^	0.0	ND^A^	0.0	ND^A^	0.0	ND^A^	0.0	ND^A^	0.0
66	ND^A^	0.0	ND^A^	0.0	ND^A^	0.0	ND^A^	0.0	ND^A^	0.0	ND^A^	0.0
77	ND^A^	0.0	ND^A^	0.0	ND^A^	0.0	ND^A^	0.0	ND^A^	0.0	ND^A^	0.0
88	ND^A^	0.0	ND^A^	0.0	ND^A^	0.0	ND^A^	0.0	ND^A^	0.0	ND^A^	0.0
99	ND^A^	0.0	ND^A^	0.0	ND^A^	0.0	ND^A^	0.0	ND^A^	0.0	ND^A^	0.0

SE, standard error.

ABCD different superscript letters denotes statistical difference (*P* < 0.05) between all treatment combinations at a given time (*t* = 0–99 days).

ND, not detected.

**Table 7.** Changes in the concentration of *C. gasigenes* (from the mean inoculation level of 2.1 log_10_ CFU/g), during the ensiling process with the different treatments.

	NS		NSFA		NSS		S		SFA		SS	
Time (d)	*C. gasigenes* (log_10_ CFU/g)											
	Mean	SE	Mean	SE	Mean	SE	Mean	SE	Mean	SE	Mean	SE
11	5.4^A^	1.2	ND^C^	0.0	0.9^BC^	0.4	1.6^B^	0.4	ND^C^	0.0	1.5^BC^	1.1
22	0.4^C^	0.2	1.7^C^	0.1	6.7^A^	0.1	3.6^B^	0.1	2.1^BC^	0.7	3.1^BC^	1.5
33	1.0^B^	0.7	1.6^B^	0.0	1.0^B^	0.4	2.0^B^	1.5	1.2^B^	0.6	3.8^A^	0.5
44	1.3^A^	0.5	ND^A^	0.0	0.4^A^	0.3	ND^A^	0.0	ND^A^	0.0	1.1^A^	0.2
55	1.1^A^	0.9	1.4^A^	0.8	1.3^A^	0.6	0.8^A^	0.5	0.5^A^	0.6	1.1^A^	0.0
66	0.9^A^	0.1	1.5^A^	0.4	1.5^A^	0.4	1.2^A^	0.0	1.5^A^	0.4	1.2^A^	0.7
77	0.8^B^	0.2	1.2^AB^	0.5	1.2^AB^	1.0	0.8^B^	0.2	2.5^A^	0.8	1.7^AB^	1.0
88	1.7^A^	0.7	1.8^A^	0.8	1.9^A^	0.7	1.6^A^	0.8	1.8^A^	0.8	1.1^A^	0.1
99	1.3^A^	0.2	1.3^A^	0.2	1.3^A^	0.2	1.2^A^	0.2	1.1^A^	0.2	1.0^A^	0.1

SE, standard error.

ABCD different superscript letters denotes statistical difference (*P* < 0.05) between all treatment combinations at a given time (*t* = 0–99 days).

ND, not detected.

## DISCUSSION

Previous research has shown that silage, a commonly used winter feed for ruminants in many countries (Driehuis *et al*. 2018) may be contaminated with *C. estertheticum* and *C. gasigenes* (Esteves *et al*. 2020). However, whether this was due to post ensiling cross contamination or survival during the ensiling process was unknown. The aim of this study was therefore to investigate the survival of *C. estertheticum* and *C. gasigenes* during the ensiling of grass, taking current practices, such as the application of slurry amendments to promote grass growth pre-harvesting and the addition of formic acid or sucrose to promote rapid preservation, into account.

Although the pH and chemical composition of the grass silage samples were generally within the limits described by Kung *et al*. (2018), the relative concentrations of lactic acid versus acetic acid and ethanol suggest our fermentation may not be representative of typical grass silage fermentations (O'Kiely and Wilson 1991; Nyambe *et al*. 2017). Interestingly the highest level of ethanol was found in fermentations when sucrose was added suggesting the presence and activity of epiphytic yeast which converts sucrose to ethanol, although some LAB species may convert lactic acid to acetic acid and ethanol (Driehuis and van Wikselaar 2000; Kung *et al*. 2000; Krooneman *et al*. 2002). The observed increase in pH and decrease in lactic acid after opening the laboratory silo tubes is also commonly observed when silage pits or bags are opened on farms (Östling and Lindgren 1991). The low levels of acetic, butyric, propionic acids and ammonia obtained in our silage samples was not sufficient to preserve the grass after opening, providing further evidence that a good quality silage requires both acetic and propionic acid to ensure stability of the silage once exposed to the air (Langó and Heinonen-Tanski 1995).

In our study the TVC count was relatively stable (approximately 6.0 +/– 1.0 log_10_ CFU/g) throughout the fermentation. *Enterobacteriaceae* and *E. coli* did not survive the ensiling process with reductions of approximately 6.0–7.0 .0 log_10_ CFU/g obtained. These findings are consistent with those previously reported by our research group, who used a similar laboratory silo system and silage treatment combinations to investigate the survival of *E. coli* C600*φ*3538(Δvtx_2_::cat) and reported a stable TVC (approximately 7.0 +/– 1.0 log_10_ CFU/g) while observing reductions in TEC and ECC of 6.4 and 6.2 log_10_ CFU/g, respectively (Nyambe *et al*. 2017). Byrne and her team also reported that *Enterobacteriaceae* and *E. coli* do not survive the grass ensiling process (Byrne *et al*. 2002). These observed reductions during the ensiling of grass are most likely due to the synergistic, antimicrobial relationship between the reduction in pH, the accumulation of organic acids (produced by the LAB) and other compounds produced during the fermentation as well as the anaerobic conditions (Lindgren 1986; Heron, Wilkinson and Duffus 1993; Russell and Diez-Gonzalez 1997).

LAB are responsible for fermenting the grass during the ensiling process and as expected increased from approximately 4.0 to 8.0 log_10_ CFU/g after 11 days. Levels of LAB showed subsequent decrease of 2.0–4.0 log_10_ CFU/g by 99 days, regardless of treatment. A similar LAB population, which ranged from 5.2 to 7.1 log_10_ CFU/g during a 100 days silage fermentation, has been reported (Nyambe *et al*. 2017).

Slurry, a commonly used fertilizer that may serve as a source of *Clostridium* spp. on grass harvested for ensiling (Bagge, Persson and Johansson 2010; Manyi-Loh *et al*. 2016; Usui *et al*. 2017), did not affect the survival of the target BPS *Clostridium* spp. In theory, the production of lactic acid and resultant drop in pH, inhibit the germination and growth of *Clostridium* spores. This study suggests that the application of formic acid or sucrose treatments did not affect the survival of *C. estertheticum* or *C. gasigenes*, although previous research had suggested that creating conditions favouring LAB could indirectly inhibit *Clostridium* spp. (Winters, Fychan and Jones 2001).

Overall, *C. gasigenes* survived the ensiling process used in this study while *C. estertheticum* were not detected by day 44. Although data is limited, previous studies have reported that *Clostridium* spp. usually survive a range of fermentation processes. *Clostridium botulinum* and *Clostridium perfringens* spores, for example, survive during anaerobic digestion (Chauret, Springthorpe and Sattar 1999; Jones and Martin 2003; Fröschle *et al*. 2015) while *Clostridium difficile* spores survive during manure composting (Rodriguez *et al*. 2019). The survival of *Clostridium* spores in baled silage has also been reported (Jonsson *et al*. 1990), while previous studies have demonstrated the survival of *Clostridium tyrobutyricum* during the ensiling process (Vissers *et al*. 2007; Li *et al*. 2020).

Difference in susceptibilities during silage fermentations may be due to *C. gasigenes* being a more robust organism, indirect evidence for which comes from its much higher prevalence (4- to 25-fold) on Irish farms (Esteves *et al*. 2020). Differences in the temperature growth range for these two bacteria may also be a factor. *C. estertheticum* has a temperature growth range of –2 to 22°C with an optimum of 6–15°C (Yang, Gill and Balamurugan 2010) while the equivalent figures for *C. gasigenes* are –1.5 to 26°C and 20–22°C (Broda *et al*. 2000). In our study, where a storage temperature of 20°C was used, *C. gasigenes* concentrations increased up to 5.4 (NS), 6.7 (NSS), 3.6 (S) and 3.8 (SS) log_10_ CFU/g. In contrast, there was no observed increase in the *C. estertheticum* population. Thus, as the fermentation proceeded the 2–3 log_10_ CFU/g reduction in BPS *Clostridium* spp. was sufficient to eliminate *C. estertheticum* while leaving a residual *C. gasigenes* population (approximately 1.0 log_10_ CFU/g). However, this would not explain the survival of *C. gasigenes* in the mini silos packed with grass treated with formic acid. No growth was observed as rapid acidification reduced the pH to 5.3 from 5.4 which is at or just below the growth limit (Broda *et al*. 2000), a phenomenon previously reported (Muck, Moser and Pitt 2003). Moreover, at this pH the immediate growth of LAB is favoured, which may further account for the suppression of BPS clostridial growth (Jones *et al*. 2009; Yang, Balamurugan and Gill 2011).

Other factors may therefore be involved in the observed survival of *C. gasigenes* spores as compared to *C. estertheticum*. These may include differences in the genetic makeup of these species, sporulation conditions, composition of the spore coat, permeability to water content and concentration of minerals in the core, the saturation of spore DNA with α/β-type small, higher concentrations of acid-soluble proteins (SASP) as well as the ability to repair damage to macromolecules (Nicholson *et al*. 2000). However, further research would be required to investigate the impact of each.

## CONCLUSION

In this study, the survival of *C. estertheticum* and *C. gasigenes* in inoculated grass during the ensiling process was investigated. *C. estertheticum* was not detected after approximately 44 days while *C. gasigenes* was still present after 99 days. Further research is required to investigate possible reasons for the survival of the *C. gasigenes* spores, to identify the specific conditions minimizing their survival during grass silage preparation and to assess the impact of *C. gasigenes* contaminated silage on the probability of BPS of beef primals later in the beef chain.

## FUNDING

This study was funded by the Teagasc and the Walsh Scholarship scheme (project number: 6910NF).
